# Machine Learning for Halide Perovskite Materials ABX_3_ (B = Pb, X = I, Br, Cl) Assessment of Structural Properties and Band Gap Engineering for Solar Energy

**DOI:** 10.3390/ma16072657

**Published:** 2023-03-27

**Authors:** Afnan Alhashmi, Mohammed Benali Kanoun, Souraya Goumri-Said

**Affiliations:** 1 Department of Physics, College of Science, King Faisal University, P.O. Box 400, Al-Ahsa 31982, Saudi Arabia; 2 Department of Mathematics and Sciences, College of Humanities and Sciences, Prince Sultan University, P.O. Box 66833, Riyadh 11586, Saudi Arabia; mkanoun@psu.edu.sa; 3 Physics Department, Colleges of Science and General Studies, Alfaisal University, P.O. Box 50927, Riyadh 11533, Saudi Arabia

**Keywords:** DFT, machine learning, band gap, perovskites, solar cells

## Abstract

The exact control of material properties essential for solar applications has been made possible because of perovskites’ compositional engineering. However, tackling efficiency, stability, and toxicity at the same time is still a difficulty. Mixed lead-free and inorganic perovskites have lately shown promise in addressing these problems, but their composition space is vast, making it challenging to find good candidates even with high-throughput approaches. We investigated two groups of halide perovskite compound data with the ABX_3_ formula to investigate the formation energy data for 81 compounds. The structural stability was analyzed over 63 compounds. For these perovskites, we used new library data extracted from a calculation using generalized-gradient approximation within the Perdew–Burke–Ernzerhof (PBE) functional established on density functional theory. As a second step, we built machine learning models, based on a kernel-based naive Bayes algorithm that anticipate a variety of target characteristics, including the mixing enthalpy, different octahedral distortions, and band gap calculations. In addition to laying the groundwork for observing new perovskites that go beyond currently available technical uses, this work creates a framework for finding and optimizing perovskites in a photovoltaic application.

## 1. Introduction

Perovskite materials have recently occupied the interest of scientists and researchers in solar cells because they have a cheap fabrication cost and are simple to synthesize and are more efficient than silicon traditional cells [1,2,3,4]. As photovoltaic materials, perovskites have distinct advantages such as bandgap tunability, a high absorption coefficient, high photoluminescence (PL) quantum yield, low trap density, and low-cost solution processing [4]. Perovskite solar cells (PSCs) have received a lot of interest because of their ever-increasing power conversion efficiency (PCE), low-cost materials constituents, and simple solution production technique [5,6]. The high density of traps, which can lead to nonradiative recombination and reduced device performance, is one of the challenging axes of research recently. Various passivation techniques, such as surface engineering and defect engineering, have been used to reduce the trap density in perovskite solar cells. For example, the use of organic and inorganic passivation layers, such as alkylamines and metal oxides, can effectively passivate the trap states and improve device performance [7].

In solar and LED research, halide perovskites have recently made breakthroughs, defying conventional wisdom [4]. In addition to solar cells, halide perovskite materials have been used in light-emitting diodes, catalysts, batteries, and photodetectors, among other optoelectronic and energy devices [3]. Perovskite takes its name from the Russian mineralogist L.A. Perovski who discovered the inorganic mineral calcium titanate (CaTiO_3_) in 1839. It is now used to represent a collection of chemical compounds that have the same crystal structure as CaTiO_3_ [2]. The general chemical formula of Halide perovskites is ABX_3_, where A is an organic or inorganic cation, B is a metal cation, and X is a halide anion. By altering the cation and halide compositions, it can provide a one-of-a-kind opportunity to realize a wide range of different material properties [4]. The optimal structure of simple perovskite is often a cubic structure. Inorganic cations or tiny organic groups A occupy the eight vertices’ angles of the cube, and cations B occupy the body center position of an octahedron formed by six X anions [BX6]^4−^ located at the six central positions of faces in the cubic [2].

Perovskites are divided into two groups: inorganic oxide perovskites (ABO_3_) and halogenated perovskites, which can be inorganic or organic–inorganic (hybrids). In the structure of ABO_3_ perovskite, several materials based on complex oxides crystallize. They have played an important role in the chemistry of materials and the physics of condensed systems throughout the last several decades. Ceramic capacitors, piezoelectric, high-temperature superconductors, nonlinear materials, gigantic magnetoresistance materials, high-quality ultra-high-frequency dielectrics, ionic conductors, and multiferroics are only a few of the applications for perovskites based on the oxide system. Organic–inorganic perovskites have lately been considered as potential thin-film solar cell materials [7,8,9].

In this paper, we will focus on halide perovskites ABX_3_ (where anion X can be Chloride ‘Cl’, Bromine ‘Br’, Iodine ‘I’, and Fluorine ‘F’). To make perovskites, most elements in the periodic table can theoretically replace the A or B in ABX_3_ [2], where A denotes a monovalent alkali metal or small organic molecule and B represents a divalent ionic metal (e.g., Pb^2+^, Sn^2+^, or Ge^3+^). One of the most active materials is ABX_3_ inorganic perovskite. They come in a wide variety of shapes and sizes, and they have a wide range of material properties, including ferroelectricity and piezoelectricity. These features are unmatched by other known materials in many applications, making inorganic perovskites crucial in domains including magnetic refrigeration, photocatalysis, and solid oxide fuel cells. Most elements in the periodic table can theoretically substitute the A or B in ABX_3_ to make perovskites [2]. Methylammonium lead trihalide (which was studied by Weber for the first time in 1978) is the most common absorbent substance used in perovskite solar cells (MAPbX_3_, where X is a halide, which may be Cl, Br, or I) [7]. Carrier mobility, bandgap, grain size, exciton binding energy, and material crystallinity are all optical and electrical features of perovskite materials. The HOMO (highest occupied molecular orbital) and LUMO (lowest unoccupied molecular orbital) levels of hole transporting layers (HTLs) and electron transport layers (ETLs), as well as the HOMO/LUMO levels of hole transporting layers (HTLs) and electron transport layers (ETLs), all influence device performance [3]. The first hurdle in perovskite development and design has discovered an efficient way to define whether a compound with formula ABX_3_ displays the perovskite structure [2]. To examine the stability and distortion of the perovskite structure, one can use the tolerance factor noted t and the octahedral factor noted μ to form perovskite. The tolerance and octahedral factors are important parameters for determining perovskite structural stability and predicting novel perovskites. [8].Goldschmidt’s tolerance factor t was developed in early 1926 and has since become widely regarded as a criterion for determining the formability of perovskites, including oxides, fluorides, and chlorides [9]. The tolerance factor t is defined by the ratio of the link length (A-X) and (B-X). The tolerance factor (t) for a perovskite structure is a constant, which can be expressed as follows:

(1) $$t=\frac{\left(R_{A}+R_{X}\right)}{\sqrt{2}\left(R_{B}+R_{X}\right)}$$

where R_A_ and R_B_ and R_X_ are the ionic radii of A, B, and X, respectively. To maintain a 3D perovskite structure, experimental data suggests that t values should be between 0.81 and 1.11 (0.81 < t < 1.11). A deformed structure with rhombohedral, orthorhombic, or tetragonal symmetry can be developed if the tolerance factor is between 0.8 and 0.9; if the tolerance factor is between 0.9 and 1, an ideal cubic structure can be built. [8]. A second parameter, the octahedral factor μ, is created to examine the fit of the B site cation into the X_6_ octahedron [8]. The octahedral factor is calculated as follows:

(2) $$\mu =\frac{R_{B}}{R_{X}}$$

A perovskite structure is formed by empirically established μ values between 0.442 and 0.895. Park et al., for example, apply artificial intelligence techniques to determine the correlation between octahedral distortion and bandgap values in perovskites [3]. The band gap values are shown to be considerably affected by octahedral tilting. The rise in the band gap, which correlates to a blue shift in the UV-vis absorption spectra, is assumed to be caused by octahedral tilting. Goldschmidt’s “no-rattling” assumption successfully predicts perovskite structures with an accuracy of roughly 80% [3]. Martin et al. calculated the factors t and μ for 12 halide perovskites. [6]. It is worth noting that these two characteristics are not the only ones that go into determining the formability and stability of a perovskite structure. These criteria, however, are insufficient to ensure their thermodynamic stability; nongeometric aspects such as chemical stability and bond valence should also be taken into account [8]. In addition to the tolerance and octahedral factor, the formation energy (ΔH) of compounds could be utilized to assess perovskite stability and formability. The enthalpy of formation is a crucial parameter that defines a material’s chemical and thermodynamic stability. External agents that can destabilize a material include heat, moisture, and oxygen. The energy differences between the neighboring two layers’ HOMO (highest occupied molecular orbitals) and LUMO (lowest unoccupied molecular orbitals) correlated well with power conversion efficiencies, while a more robust descriptor considers the ΔH, HOMO, LUMO, E_g_, and ΔL for general conversion efficiencies [5]. The enthalpy of formation (ΔH) is computed by considering the following decomposition reaction: MAPbI_3_ decomposes into MAI + PbI_2_. The formation energy of mixed halide perovskites was calculated using the next equation: $\Delta H=E\left({\mathrm{MA}\;\mathrm{Pb}\;I}_{3}\right)-E\left(\mathrm{MAI}\right)-E\left({\mathrm{PbI}}_{2}\right)$. Exothermic and endothermic reactions are denoted by a negative and positive ΔH, respectively. As a result, an exothermic reaction shows that the environment is more stable. The A-site cations disordered are proposed to be involved in the stabilization of overall framework structures and the formation of ferroelectric highways, all of which are linked to outstanding electrical and optical properties [3]. Chen et al. noted that the low chemical stability of CH_3_NH_3_PbI_3_ is intrinsically unstable, and it spontaneously decomposed into organic and inorganic parts, and it was suggested that element substitution could help overcome the chemical stability problem in hybrid halide perovskite solar cells [10]. In recent years, Artificial Intelligence (AI) has sparked widespread interest all over the world. Since the 1980s, machine learning (ML) has been the essence of Artificial Intelligence. ML has become a strong technique in materials science for assisting in the design and screening of various materials. It can extract helpful knowledge from existing data, including failed experiments. It can also be used to predict new experiments to optimize a specific material or even to discover new materials using parameter exploration. An algorithm can learn from the traits combined with the results of the tests, and this is used to forecast which additional tests will be most informative [11]. Machine learning has had a number of accomplishments, including applications in photovoltaic materials, superconductors, and high entropy alloys [4]. ML has been widely utilized in perovskite materials. New perovskite compositions are produced experimentally based on ML guidance to verify the model’s viability. The machine learning model also demonstrates its capacity to predict both underlying physical processes and perovskite solar cell performance. The perovskite solar cell model closely resembles the theoretical Queisser and Shockley limit prediction, which is nearly impossible for a human to identify from a set of data points. Likewise, the model yields strategies for creating high-performing perovskite solar cells with various bandgaps. These findings suggest that machine learning holds great promise not only for forecasting performance but also for offering a better understanding of the physical phenomena that accompany it [12]. The synthetic creation of dimensionally customized halide perovskites is also aided using machine learning technology. To further enhance halide perovskite-based devices, the ML technique helps the selection of components such as additives that reside on the surfaces of halide perovskites. Descriptors, datasets, lead-free halide perovskites, other halide perovskites, dimensional tailoring, stability, and additives are all covered in research studies of halide perovskite material searches using machine learning techniques. The “descriptors” and “dataset” sections outline the essential modules of ML approaches specialized for halide perovskite materials; for example, correct descriptor selection in the stage of feature engineering can increase the stability of halide perovskite materials. Both optoelectronic performance and stability gain from the dimensional tailoring process [3]. The ML process requires the identification of correct features that have a strong connection to the targeting attributes. During the feature engineering process, the features, or descriptors, can be chosen. Once the halide perovskite materials’ input and output data have been quantitatively represented, the mapping between input and output data can be conducted using the ML technique [3]. In the present work, we used density functional theory results, mainly band gap and energies, to study a family of 81 perovskites. We investigated two groups of halide perovskite compound data with the ABX_3_ formula, which are the formation energies data for 81 compounds and the stability data for 63 compounds. We used machine learning techniques by WEKA and MATLAB programs for clustering, classification, and analyzing data to study the formation, physical, and stability properties of the perovskite.

## 2. Materials and Methods

### 2.1. DFT

**Figure 1:**
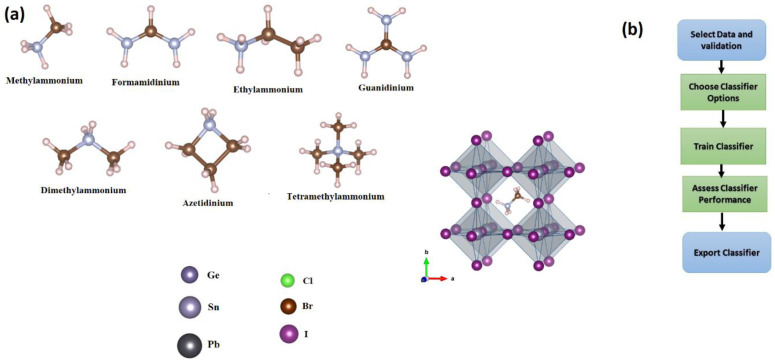
(**a**) Schematic representation of ABX_3_ with different molecules, metals, and halide atoms. Carbon, nitrogen, and hydrogen atoms are illustrated in dark brown, gray, and light pink, respectively. (**b**) Flow chart of a typical procedure for training classification models, or classifiers, in the Classification Learner program.

The dataset of hybrid perovskites was constructed by considering the seven most common organic cations, named methylammonium (MA^+^), formamidinium (FA^+^), dimethylammonium (DMA^+^), guanidinium (GUA^+^), ethylammonium (EA^+^), tetramethylammonium (TMA^+^), and Azetidinium (Az^+^), all of which have been considered in the literature [8,9,10,11]. Each of these seven cations, shown in Figure 1a, is placed at site A of the ABX_3_-based perovskites in the cubic phase. We expand the set of 63 structures by substituting either Pb or Sn or Ge for the B-site, and, similarly, by replacing either I or Br or Cl for the halide site. We also extend our dataset to 81 prototypical structures by considering only the MA^+^ cation in ABX_3_ in tetragonal and orthorhombic phases. A total 81 halide perovskites compounds are investigated.

All calculations were performed at the generalized-gradient approximation within the Perdew–Burke–Ernzerhof (PBE) [13] functional based on the density functional theory framework as implemented in the Quantum Atomistix ToolKit (QuantumATK) package [14]. The numerical linear combination of atomic orbitals (LCAO) methodology is also used. For defining the interaction between ion nuclei and valence electrons, the norm-conserving PseudoDojo [15] pseudopotential was adopted. The self-consistent field (SCF) computation was repeated until the total energy difference was less than 10^6^ Ha. All compounds are relaxed using the limited-memory Broyden–Fletcher–Goldfarb–Shanno (LBFGS) algorithm until the energies and forces are converged within 0.05 eV/Å [16]. The dataset building of roughly eighty hybrid halide perovskite systems using DFT calculations at the PBE level offers an excellent compromise between computational cost and satisfactory experimental band gap estimates.

### 2.2. Machine Learning

#### 2.2.1. MATLAB: Classification Learner App

Classification Learner allows you to interactively explore your data, pick characteristics, create validation schemes, train models, and evaluate outcomes, among other supervised learning activities. Decision trees, support vector machines, discriminant analysis, logistic regression, naive Bayes, closest neighbors, ensembles, kernel approximation, and neural networks may all be trained with Classification Learner. It may also examine the data, establish validation schemes, pick features, and assess findings in addition to training models. To learn about programmatic classification, we may export a model to the workspace and apply it with new data or produce MATLAB [17]. The process of training a model in Classification Learner is divided into two parts [17]:

**Validated Model:** Use a validation strategy to train a model. Cross-validation is used by default to prevent overfitting. We also have the option of using holdout validation. The app displays the verified model.
**Full Model:** Without validation, a model is trained on full data. This model is being trained at the same time as the verified model. Nevertheless, the software does not show the model that was trained on all the data. Classification Learner sends the whole model when you pick a classifier to export to the workspace.

To test the predicted accuracy of the fitted models, we choose a validation technique. Validation compares the performance of the model on fresh data to the training data and aids in the selection of the best model. Overfitting is avoided with validation. Before training any models, choose a validation scheme so that may compare all the models in session using the same method [18].

**Cross-Validation:** This approach provides a reasonable assessment of the prediction accuracy of the final model trained using all available data. It necessitates several fits yet efficiently utilizes all the data, making it ideal for smallish datasets. To split the dataset, choose a number of folds (or divisions).
**Holdout Validation:** The program uses the training set to train a model and the validation set to measure its performance. Because the validation model is only based on a fraction of the data, holdout validation is only advised for big datasets. The complete dataset is used to train the final model. To utilize as a validation set, choose a proportion of the data.
**Re-substitution Validation:** The program trains on all the data and computes the error rate using the same data. You obtain an inflated estimate of the model’s performance on fresh data if there is no separate validation data. That is, the accuracy of the training sample is likely to be unreasonably high, while the predicted accuracy is likely to be lower. There is no safeguard against overfitting.

The verified model’s findings are displayed in the app. The validated model findings are shown in diagnostic metrics such as model accuracy and graphs such as a scatter plot or the confusion matrix chart. You may determine the best model for your classification challenge by automatically training one or more classifiers, comparing validation results, and selecting the best model. **Classification Learner** sends the whole model when you pick a model to export to the workspace. There is no lag time when you export the model since the Classification Learner builds a model object of the whole model during training. The exported model may be used to create predictions based on new data [17,18]. To train and evaluate classification models for binary or multiclass issues, choose from a variety of algorithms. After training many models, compare their validation errors side by side and pick the best one. The flow chart in Figure 1b depicts a typical procedure for training classification models, or classifiers, in the Classification Learner program [19].

#### 2.2.2. Clustering in WEKA

The cluster window in WEKA is used to become familiar with the process of choosing and configuring items. It is used if there is no output in the data (unsupervised ML) and we need to cluster and study data. By using the cluster mode box, it can choose what to cluster and how to assess the results. The first three selections are identical to the classification options: Use training set, supplied test set, and percentage split, but instead of trying to predict a single class, the data are instead assigned to clusters. The fourth setting: Evaluation of Classes to Clusters analyzes how well the selected clusters match up with a pre-assigned class in the data. The class is selected using the drop-down box underneath this choice, exactly as it is in the Classify panel [20].

## 3. Results and Discussions

Our database including seven A cations (MA, FA, DMA, TMA, EA, GUA, AZ), three groups of B cations (Pb, Sn, Ge), and three halide anion X (I_3_, Br_3_, Cl_3_) was created using density functional theory (DFT). We entered data into both the MATLAB and WEKA programs. Then, we used some machine learning techniques for clustering, classification, and analyzing data to study the formation, physical, and stability properties of the perovskite.

### 3.1. Formation Energy and Structural Stability

**Figure 2:**
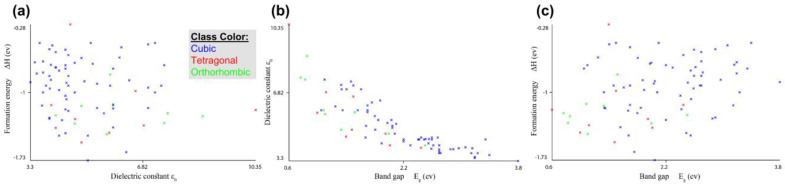
Formation energy and structural stability: the relationship between attributes obtained using WEKA. (**a**) Formation energy vs dielectric constant, (**b**) Dielectric constant vs band gap and (**c**) formation energy vs band gap.

**Figure 3:**
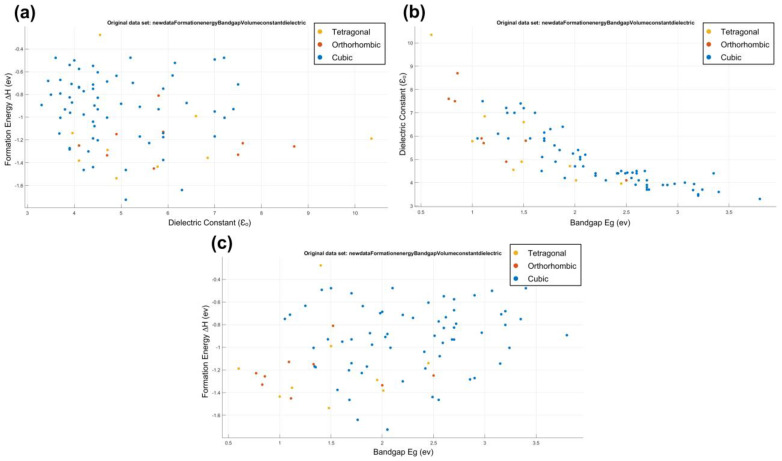
Formation energy and structural stability: the relationship between attributes obtained using MATLAB. (**a**) Formation energy vs dielectric constant, (**b**) Dielectric constant vs band gap and (**c**) formation energy vs band gap.

The formation data consist of 81 compounds of the halide perovskite formula (ABX_3_) as shown in Table S1 in the supplementary information. There are six attributes of compounds (the formula, formation energy (ΔH), volume (V), band gap (E_g_), dielectric constant (ε_0_), and structure of atoms). From these data, we can study the formation, electronic, bandgap, and physical properties of perovskite. First, we put the data into the WEKA and MATLAB programs to analyze and explain the relationship between attributes, as shown in Figure 2 and Figure 3.

As shown in Figure 2 and Figure 3, the compounds of the cubic structures have the highest values of band gap and formation energy but have the lowest value of the dielectric constant. The TMAGeCl_3_ compound has the highest value of the band gap (E_g_ = 3.8 eV) and the lowest value of the dielectric constant (ε_0_ = 3.3). In contrast, the MASnI_3_ compound has the highest dielectric constant (ε_0_ = 10.35) and the lowest band gap (E_g_ = 0.6 eV). For formation energy, the highest value is for the MASnCl_3_ compound (ΔH = −0.276591335) as for the tetragonal structure. The MAGeBr_3_ compound has the lowest value (ΔH = −1.726001626) in the cubic structure. We conclude that the relationship between the band gap and dielectric constant is an inverse relationship. The increase in the value of the dielectric constant corresponds to the decrease in the value of the band gap [21,22,23,24,25]. In fact, in a high-symmetry structure such as cubic, the electronic charge distribution is very uniform, leading to weak polarization effects and a low dielectric constant. This is because there is no preferred direction for the electronic charge to accumulate, and the crystal lattice is not easily polarizable. As a result, cubic compounds tend to have low dielectric constants compared to other crystal structures.

#### 3.1.1. Data Clustering

**Figure 4:**
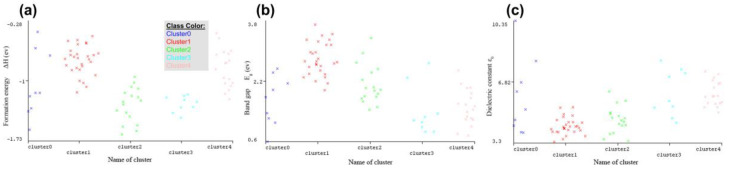
Clustering following the physical property obtained in WEKA. (**a**) Formation Energy; (**b**) Band gap; (**c**) Dielectric constant.

We used the WEKA program to cluster the data. We used the KNN algorithm, which is called SimpleKMeans. When we put 5 numbers of clusters and 11 seeds together, we obtained a good sum of squared errors of 80.95. The first cluster (*Cluster0*) contains nine elements with a similarity rate of 11%. *Cluster1* contains 28 elements with a similarity rate of 35%. *Cluster2* contains 17 elements with a similarity rate of 21%. *Cluster3* contains nine elements with a similarity rate of 11%. Finally, *Cluster4* contains 18 elements with a similarity rate of 22%. We have reported the clustering results in the supplementary information (Section 2). The WEKA cluster visual plots are shown in Figure 4.

As shown in Figure 4 and the cluster information, we can find some information for attributes:

**For ΔH**, compounds in *Cluster1* have the highest value, while compounds in *Cluster2* have the lowest value. 
**For E_g_**, the highest values are for compounds in *Cluster1*, but the lowest values are in *Cluster3*. 
**For ε_0_**, compounds in *Cluster4* have the highest values, but the lowest values are in *Cluster1*. 

The structures of clusters 1, 2, and 4 are cubic. *Cluster0* and *Cluster3* have the tetragonal and orthorhombic structure, respectively.

#### 3.1.2. Data Classifying

**Figure 5:**
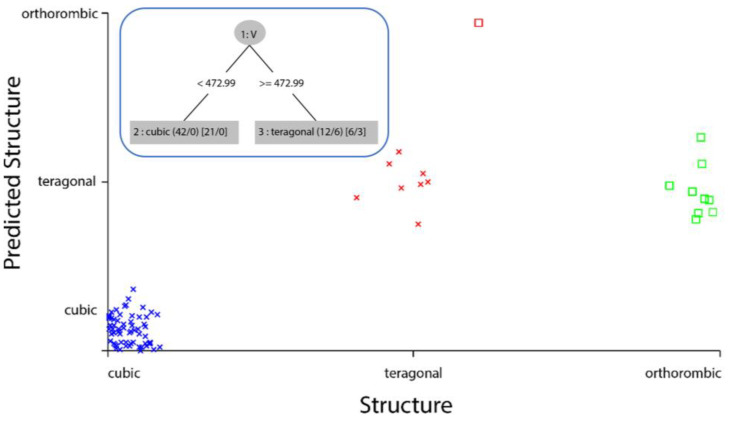
trees.REPTree classifier error visualization, where the cubic points are the error-classified structures (Inset: The tree from **trees.REPTree** classifier).

To make a classification of data, we used both WEKA and MATLAB programs and then compared the results between each. At first, we used the WEKA program to test some classification algorithms on the data. We obtained the best accuracy with the (**trees.REPTree**) classifier, with an accuracy of 87.65%. For the Kappa statistic, which is the percentage decrease in errors resulting from random classification [20], we got a ratio of 0.6667. The Root Mean Squared Error (RMSE) has a good value of 0.1973, which is a measure of how accurately the model predicts the response. [4]. The classification results are shown in the supplementary information (Section 3). As shown in the classification results in Supplementary Material S3, in the confusion matrix of the classifier, all 63 cubic structures have been correctly classified. In contrast, the tetragonal was incorrectly classified once as an orthorhombic. All nine orthorhombic were incorrectly classified as tetragonal. The tree and visualization classifier errors of (**trees.REPTree**) classifiers are shown below in Figure 5, in which the cubic points are the error-classified structures.

**Figure 6:**
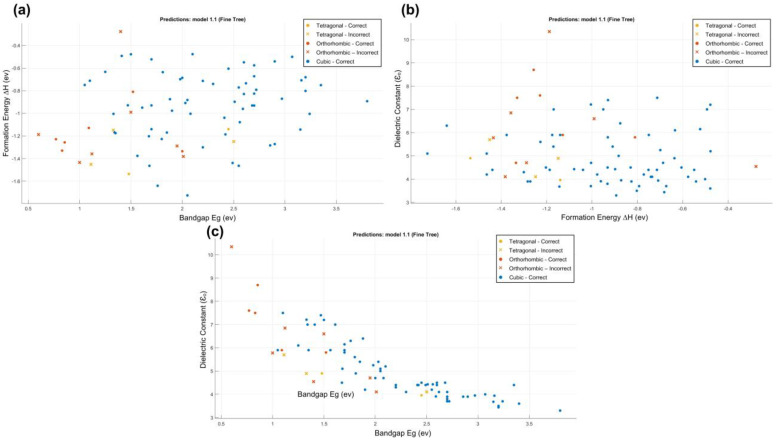
The classification error representation of the tree classifier, where x points represent the incorrect classification of data. (**a**) Formation Energy; (**b**) Band gap; (**c**) Dielectric constant.

**Figure 7:**
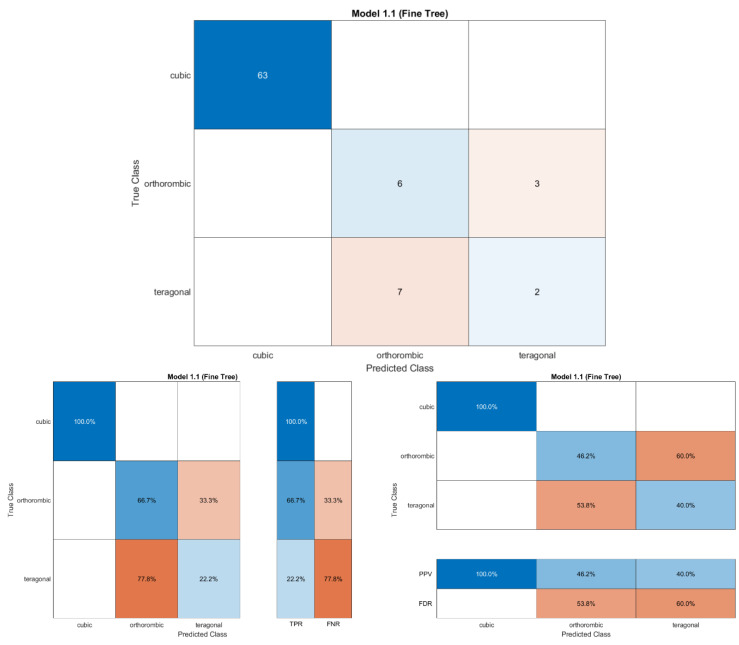
The confusion matrix, TPR and FNR matrix, and PPV and FDR matrix.

Secondly, we used the MATLAB program to classify data and enter the programming commands model to filter data in photovoltaic bandgap ranges. First, in MATLAB, we used the **Classification Learner App** to analyze and interpret data. When we trained all the classifiers, the tree classifiers had the best accuracy with 87.7%, which is an approximation similar to that in WEKA. The classification errors are shown in Figure 6 and Figure 7, where the x points represent the incorrect classification data.

From the confusion matrix of the tree classifier (Figure 7), it can be shown that all cubic structures were predicted correctly, but the orthorhombic and tetragonal were wrongly predicted. The classifier predicted the orthorhombic three times as tetragonal structures and the tetragonal two times as orthorhombic structures. The true positive rate (TPR) predicted for the cubic is 100%, but the TPR predicted is 66.7% for the orthorhombic and 22.2% for the tetragonal. After classifying the data, we used a programming commands model to filter the data in a range of photovoltaic band gap (1.1 < Eg <1.6), as reported in the supplementary information (Supplementary Material S4). Table 1 shows the compounds that have a good range of photovoltaic bandgap values, extracted from the main database. We conclude that the formulas with an anion MA (MABX_3_) have the highest number of compounds with good values of the band gap (E_g_).

For a more in-depth analysis, we divided the data into train and test data, with the test data accounting for 20% of the total, and then classified them, and finally made the classification of the train and test data. When we tested all the classifiers in the **Classification Learner App** (see Supplementary Material S4), we obtained the best accuracy with the **Fine Tree** and the **Kernel Naïve Bayes** classifier, with an accuracy rate of 84.6%. The data splitting model is illustrated in the supplementary information.

### 3.2. Stability of Structure: Clustering and Classification of Data

For the stability data, the data consists of 63 compounds of the perovskite formula (ABX_3_) as shown in Table S2 in the supplementary information (Supplementary Material S5). These data contain three attributes: the formula of a compound, the tolerance factor t, and the octahedral factor μ. We used unsupervised machine learning on this dataset because we did not have any output. First, we used the WEKA program to cluster the stability data, and then we used classification in both programs, WEKA and MATLAB, to study and simulate data. As a first step, we used the WEKA program to cluster the stability data into groups to visualize it and find the relationship between t and μ, because we do not have any output in the data. We applied the **SimpleKMeans** algorithm to cluster data, and we put 5 numbers of clusters and 11 seeds from the algorithm setting to obtain a good sum of square errors of about 58.98. The first cluster (*Cluster0*) contains six elements with a similarity rate of 10%. *Cluster1* has five elements with a similarity rate of 8%. *Cluster2* has 18 elements with a similarity rate of 29%, and *Cluster3* has 16 elements with a similarity rate of 25%. Finally, *Cluster4* contains 18 elements with a similarity rate of 29%. We have shown the clustering results in the supplementary information (Supplementary Material S6).

**Figure 8:**
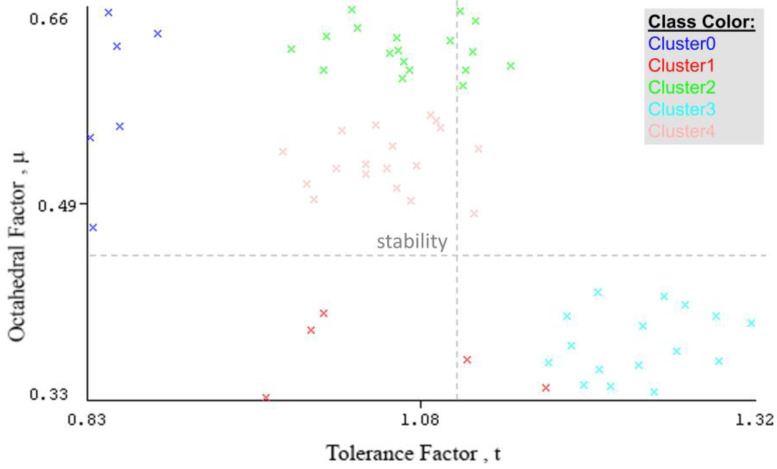
Values of tolerance and octahedral factors based on cluster number.

As shown in Figure 7, values in *Cluster3* are within an instability limit for the tolerance and octahedral factors (where the stability limits are 0.81 < t < 1.11 for the tolerance factor, and 0.44 < μ < 0.89 for the octahedral factor). Compounds in *Cluster0*, *Cluster2*, and *Cluster4* have approximately stable values for both tolerance and octahedral factors. Figure 8 shows the values of tolerance and octahedral factors based on cluster number after using the filter (add cluster) in WEKA to add the number of clusters as an extra category in the stability database.

**Figure 9:**
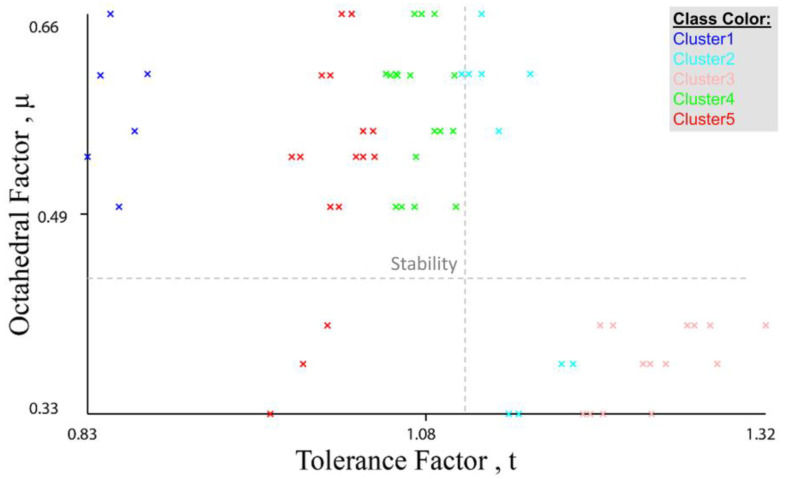
Octahedral factor vs. tolerance factor.

**Figure 10:**
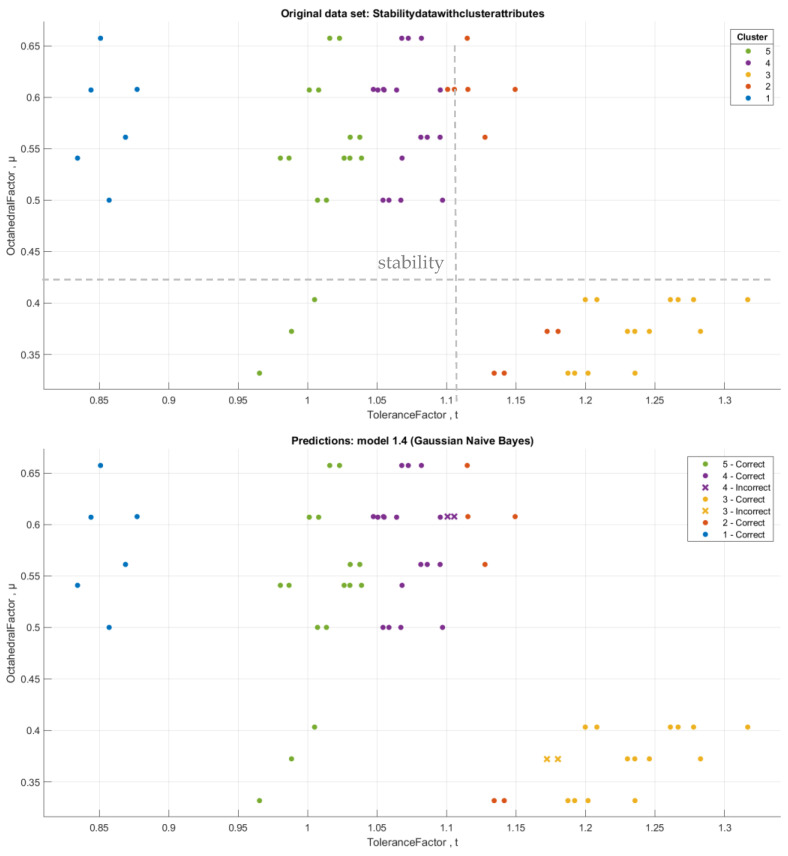
The classification error representation of the Gaussian Naive Bayes classifier, where x points represent the incorrect classification of data.

**Figure 11:**
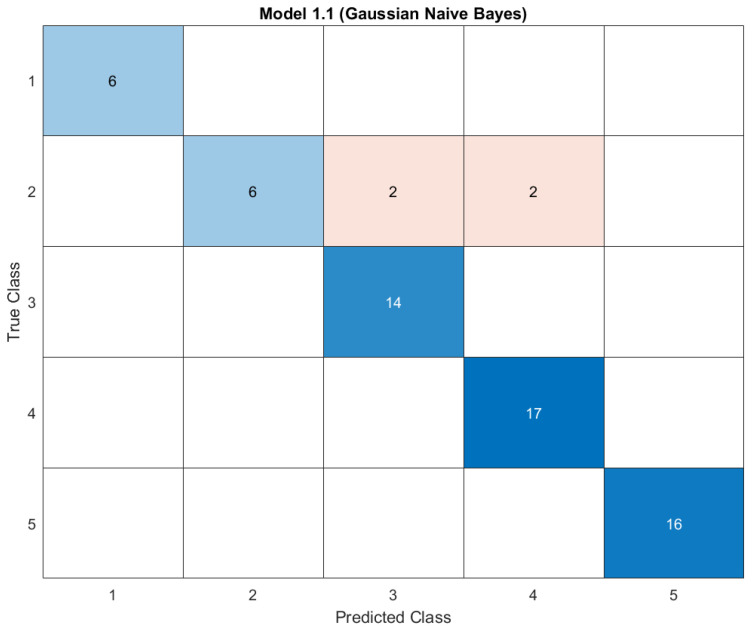

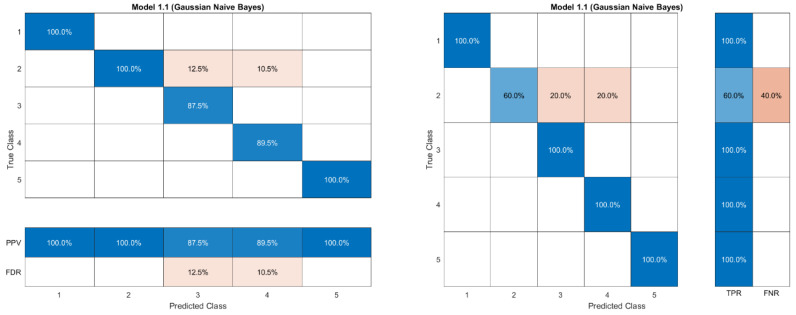
Confusion matrix and TPR and FNR matrices of the Gaussian Naive Bayes classifier.

We used the filter (add cluster) in WEKA to add the number of clusters as an extra category in the stability database. Table S3 in the supplementary information (S7) illustrates the database with a new attribute, which is the cluster. From Figure 9, it is clear that compounds in *Cluster 2* and *Cluster 3* are in the instability limits for both the tolerance and octahedral factors (0.81 < t < 1.11, 0.44 < μ < 0.89). All compounds in *Cluster1*, *Cluster 4*, and *Cluster5* are within the stability limits of the tolerance factor. After we added the cluster category, we used the classification of data using the WEKA program, followed by the MATLAB program. In WEKA, we used some different algorithms, and we obtained the best accuracy with the **trees.j48** and **functions.Logistic** classifiers, with an accuracy rate of 93.65% and 95.23%, respectively. The run information results for the **trees.j48** and **functions.Logistic** classifiers are illustrated in the supplementary information (S8 and S9 and Figures S1 and S2). On the other hand, when we used the Classification Learner App in the MATLAB program to classify the new data with the cluster attribute, we found the best accuracy for the **Gaussian Naive Bayes** classifier reached 93.7%, which is a similar accuracy rate to the tree classifier in WEKA. Figure 10 and Figure 11 show the visualization of data, the classification errors, and the classifier confusion matrix using MATLAB.

Figure 11 shows the confusion matrix of the **Gaussian Naïve Bayes** algorithm, which represents the True Positive Rate (TPR) and False Negative Rate (FNR) of the predicted class. All clusters were correctly predicted except *Cluster2*. Four compounds in *Cluster2* were incorrectly predicted; two of them were predicted as compounds in *Cluster3* and two as compounds in *Cluster4*. We coded a short program model in MATLAB using the conditions of the stability limits to identify the stable compounds; “non” if all factors are in the instability limits, “one stable” if only one factor is in the stability limits, and “stable” if both t and μ factors are in the stability limits. The script of the model is reported in the supplementary information (Supplementary Material S10).

**Figure 12:**
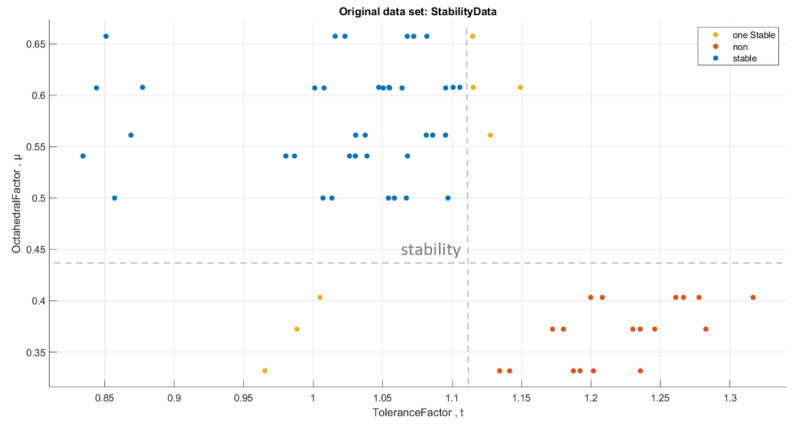

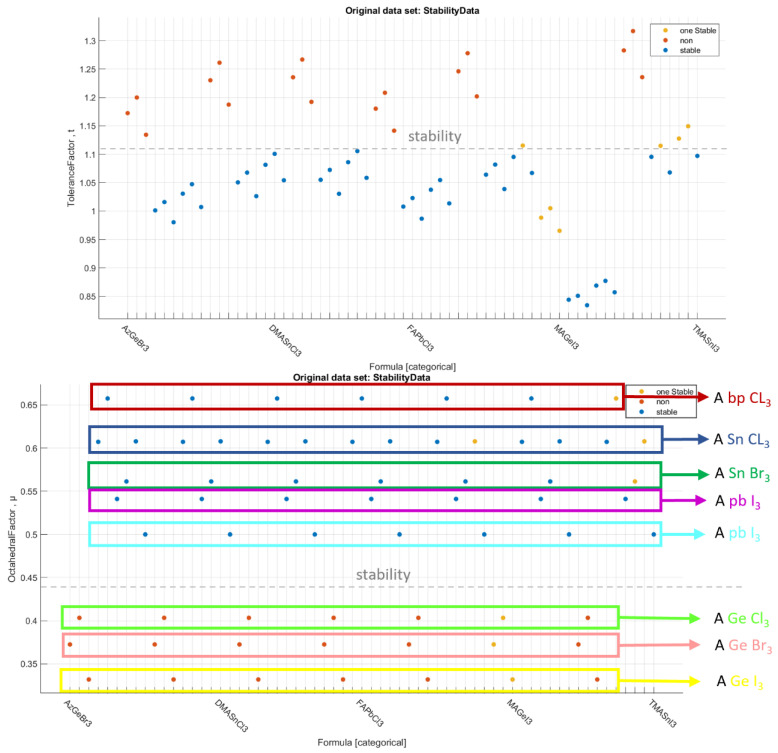
Stability data analysis of the perovskite compounds (ABX_3_).

Figure 12 shows the stability data analysis of the perovskite compounds (ABX_3_) 

**Figure 13:**
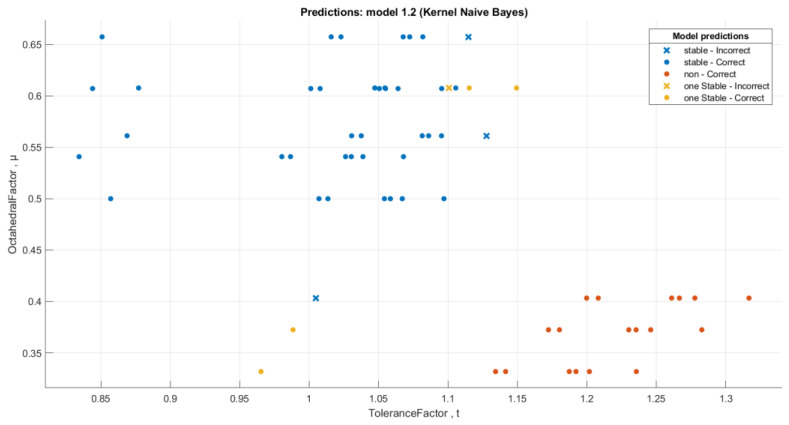
The Kernel Naive Bayes classifier prediction model for the data with stability conditions.

**Figure 14:**
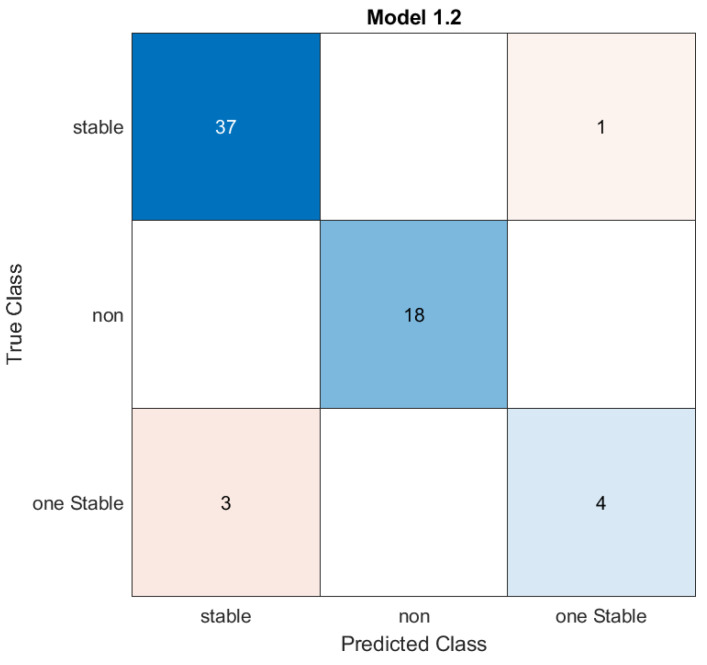
The confusion matrix of the Kernel Naive Bayes classifier for the data with stability conditions.

We performed a classification of the data with stability conditions using the **Classification Learner App** in MATLAB. When we used all classifiers, the best accuracy was for the **Kernel Naive Bayes** classifier [26,27,28] with an accuracy rate of 93.7%. Figure 13 and Figure 14 below show the visualization of the prediction errors and confusion matrix of the classifier.

Table S4 (supplementary information, Supplementary Material S11) shows the final 38 compounds extracted from the main dataset that are within the stability limits of the t and μ factors. Finally, we made a classification of the final 38 stability data using the **Classification Learner App** in MATLAB. When we used all classifiers, the best accuracy was for the **Kernel Naive Bayes** classifier, with an accuracy rate of 94.7%. The Figures S3 and S4 in the supplementary information (Supplementary Material S11) below show the visualization of the prediction errors and confusion matrix of the classifier.

## 4. Conclusions

In summary, we have reviewed in detail perovskite solar cells and their structural, thermal, chemical, electronic, and optical properties. In addition, we reviewed the challenges and problems of using halide perovskite in solar cells, such as formation and stability issues under different environmental conditions such as moisture, UV irradiation, increased temperature, and exposure to the ambient atmosphere. We have shown that hundreds of perovskite compositions may be examined at high-speed using machine learning techniques, which save a significant amount of time and effort compared to experimental methods. There are some previous studies on perovskite and the use of machine learning to classify and analyze perovskite cells. In this paper, we utilized machine learning techniques using MATLAB and WEKA programs to study the data of 81 perovskite compounds and identify the optimal bandgap values for each compound as well as the most stable one. We may conclude that the cubic structure compounds have the highest band gap and formation energy but the lowest dielectric constant values. When we used the classification on the data, we got the best accuracy in the tree classifier, with 87% for formation data and 93% for stability data. We extracted 16 compounds from the formation database that have a band gap value in the range of the photovoltaic band gap (1.1 < E_g_ <1.6) by creating a small program to filter data. Formulas with an anion MA (MABX_3_) were shown to have the greatest number of compounds within the band gap ranges (E_g_). Furthermore, we made a small program model in MATLAB for stability data using the conditions of the stability limits to identify the stable compounds. We deduced that the B-cation, which has a germanium element (Ge) in the halide perovskite materials (ABX_3_), is in the unstable limits of the octahedral and tolerance factors. By creating a program model in MATLAB using the stability dataset, we extracted 38 compounds that are within the stability limits of the octahedral and tolerance factors. In conclusion, the importance of using machine learning and artificial intelligence techniques in material science, especially in perovskite materials, is to achieve impressive results in designing new materials with high efficiency with less cost and effort. In the future, machine learning is expected to become an essential complementary tool for experiments and calculations in the field of materials research.

## Figures and Tables

**Table 1:** The best 16 compounds that have the appropriate range of photovoltaic bandgap values: (1.1 eV < E_g_ <1.6 eV).

*Formula*	Δ*H* (eV)	*V (Å*^3^)	*E*_*g*_ (eV)	*ε*_0_	*Structure*
‘MASnI_3_’	−1.3763	231.2129	1.563	5.9	‘cubic’
‘MAPbI_3_’	−0.9900	956.5107	1.5	6.6	‘tetragonal’
‘MASnCl_3_’	−0.2766	738.4159	1.4	4.55	‘tetragonal’
‘MAGeI_3_’	−1.3580	786.5942	1.12	6.85	‘tetragonal’
‘MAGeBr_3_’	−1.5365	753.4887	1.48	4.9	‘tetragonal’
‘MAPbI_3_’	−0.8101	919.0582	1.52	5.8	‘orthorhombic’
‘MASnBr_3_’	−1.4514	779.5246	1.11	5.7	‘orthorhombic’
‘MASnCl_3_’	−1.1491	681.0253	1.33	4.9	‘orthorhombic’
‘FAPbI_3_’	−0.4778	250.7740	1.5	7.2	‘cubic’
‘DMAPbI_3_’	−0.4925	259.4683	1.41	7	‘cubic’
‘DMASnI_3_’	−0.7121	251.1712	1.1	7.5	‘cubic’
‘TMASnI_3_’	−0.6336	286.3171	1.25	6.1	‘cubic’
‘EASnI_3_’	−1.1689	249.8638	1.34	7	‘cubic’
‘GUAPbI_3_’	−0.9293	255.4561	1.47	7.4	‘cubic’
‘AZPbI_3_’	−1.0048	260.1068	1.33	7.21	‘cubic’

## Data Availability

Not applicable.

## Supplementary Materials

The following supporting information can be downloaded at: https://www.mdpi.com/article/10.3390/ma16072657/s1.
