# Supplementary material for: Machine Learning for Halide Perovskite Materials ABX3 (B = Pb, X = I, Br, Cl) Assessment of Structural Properties and Band Gap Engineering for Solar Energy
Source: Materials (Basel). 2023 Mar 27;16(7):2657. doi: 10.3390/ma16072657 (PMC10095675; doi:10.3390/ma16072657)
Supplement: Supplementary file 1 [file materials-16-02657-s001.zip › materials-2252368-supplementary.pdf]

Supplementary data

Afnan Alhashmi [1], Mohammed Benali Kanoun [2] and Souraya Goumri-Said [3,*]

[1] Department of Physics, College of Science, King Faisal University, P.O. Box 400, Al-Ahsa 31982, Saudi Arabia
[2] Department of Mathematics and Sciences, College of Humanities and Sciences, Prince Sultan University, P.O. Box 66833, Riyadh 11586, Saudi Arabia; mkanoun@psu.edu.sa
[3] Physics Department, Colleges of Science and General Studies, Alfaisal University, P.O. Box 50927, Riyadh 11533, Saudi Arabia
* Correspondence: sosaid@alfaisal.edu; Tel.: +966-112-158-984

S1. Table S1. Formation Data for 81 ABX_3 perovskites.

	Formula	*delta H (ev)*	*V*	*Eg (ev)*	*eps0*	*structure*
1	$MAPbI_3$	-0.908721326	245.03719	2.03	5.4	cubic
2	$MAPbBr_3$	-1.439176426	205.663607	2.49	4.4	cubic
3	$MAPbCl_3$	-1.28355653	181.9883	2.856	3.9	cubic
4	$MASnI_3$	-1.37631947	231.212919	1.563	5.9	cubic
5	$MASnBr_3$	-1.464300559	192.322513	1.68	5.1	cubic
6	$MASnCl_3$	-1.203087249	169.336753	1.675	4.5	cubic
7	$MAGeI_3$	-1.640658436	210.434461	1.76	6.3	cubic
8	$MAGeBr_3$	-1.726001626	179.909605	2.05	5.1	cubic
9	$MAGeCl_3$	-1.464040205	159.820718	2.55	4.2	cubic
10	$MAPbI_3$	-0.99001602	956.510671	1.5	6.6	tetragonal
11	$MAPbBr_3$	-1.288475969	805.564731	1.95	4.71	tetragonal
12	$MAPbCl_3$	-1.139852308	716.420495	2.45	3.96	tetragonal
13	$MASnI_3$	-1.187807792	934.204581	0.6	10.35	tetragonal
14	$MASnBr_3$	-1.4349335	801.567061	1	5.78	tetragonal
15	$MASnCl_3$	-0.276591335	738.415858	1.4	4.55	tetragonal
16	$MAGeI_3$	-1.35795805	786.594233	1.12	6.85	tetragonal
17	$MAGeBr_3$	-1.536461912	753.488654	1.48	4.9	tetragonal

18	$MAGeCl_3$	-1.382272633	659.666015	2.01	4.1	tetragonal
19	$MAPbI_3$	-0.81006116	919.058209	1.52	5.8	orthorhombic
20	$MAPbBr_3$	-1.335260294	797.538219	2	4.7	orthorhombic
21	$MAPbCl_3$	-1.249112467	715.195393	2.5	4.1	orthorhombic
22	$MASnI_3$	-1.22899541	915.973874	0.77	7.6	orthorhombic
23	$MASnBr_3$	-1.4513816	779.524593	1.11	5.7	orthorhombic
24	$MASnCl_3$	-1.149066142	681.025304	1.33	4.9	orthorhombic
25	$MAGeI_3$	-1.257113681	823.4845	0.855	8.7	orthorhombic
26	$MAGeBr_3$	-1.329922693	688.628935	0.83	7.5	orthorhombic
27	$MAGeCl_3$	-1.129166739	603.725147	1.09	5.9	orthorhombic
28	$FAPbI_3$	-0.477824046	250.77397	1.5	7.2	cubic
29	$FAPbBr_3$	-0.549036961	216.546682	2.6	4.4	cubic
30	$FAPbCl_3$	-1.272007144	193.517739	2.9	3.9	cubic
31	$FASnI_3$	-0.749274702	241.317051	1.05	5.9	cubic
32	$FASnBr_3$	-0.636200491	208.473339	1.81	4.9	cubic
33	$FASnCl_3$	-1.30070581	188.695049	2.2	4.3	cubic
34	$FAGeI_3$	-0.875304253	224.060664	1.88	6.4	cubic
35	$FAGeBr_3$	-0.477150368	191.102942	2.1	5.2	cubic
36	$FAGeCl_3$	-1.186501893	172.017238	2.42	4.4	cubic
37	$DMAPbI_3$	-0.492518057	259.468341	1.41	7	cubic
38	$DMAPbBr_3$	-0.60536612	214.633747	2.45	4.5	cubic
39	$DMAPbCl_3$	-0.54088092	193.871035	2.9	3.9	cubic
40	$DMASnI_3$	-0.712147201	251.171209	1.1	7.5	cubic
41	$DMASnBr_3$	-0.686408253	214.266082	2	4.7	cubic
42	$DMASnCl_3$	-0.672413639	200.942108	2.7	3.7	cubic
43	$DMAGeI_3$	-0.698507977	241.28263	1.98	5.25	cubic
44	$DMAGeBr_3$	-0.77150832	206.514052	2.55	4.2	cubic
45	$DMAGeCl_3$	-0.802250595	187.801791	3.2	3.5	cubic
46	$TMAPbI_3$	-0.522804956	285.0537	1.7	6.15	cubic
47	$TMAPbBr_3$	-0.73457816	263.814655	2.62	4.1	cubic
48	$TMAPbCl_3$	-0.478176436	236.63358	3.4	3.6	cubic

49	$TMASnI_3$	-0.633599056	286.31714	1.25	6.1	cubic
50	$TMASnBr_3$	-0.740205963	258.291765	2.3	4.1	cubic
51	$TMASnCl_3$	-0.681181433	238.382968	3.2	3.44	cubic
52	$TMAGeI_3$	-0.713486867	289.454946	2.2	4.4	cubic
53	$TMAGeBr_3$	-0.792016961	259.886552	2.72	3.7	cubic
54	$TMAGeCl_3$	-0.892964973	232.748077	3.8	3.3	cubic
55	$EAPbI_3$	-0.950075649	256.666118	1.61	7	cubic
56	$EAPbBr_3$	-0.897816927	216.197943	2.51	4.43	cubic
57	$EAPbCl_3$	-0.871243281	192.621649	2.97	3.95	cubic
58	$EASnI_3$	-1.168934793	249.863822	1.34	7	cubic
59	$EASnBr_3$	-0.97688106	212.827676	1.9	4.2	cubic
60	$EASnCl_3$	-0.960882001	193.904703	2.59	3.91	cubic
61	$EAGeI_3$	-0.930092569	230.124166	1.7	5.8	cubic
62	$EAGeBr_3$	-1.039604127	206.918648	2.41	4.4	cubic
63	$EAGeCl_3$	-1.143701956	185.060178	3.15	3.68	cubic
64	$GUAPbI_3$	-0.929288266	255.456063	1.47	7.4	cubic
65	$GUAPbBr_3$	-0.829315137	214.041833	2.6	4.5	cubic
66	$GUAPbCl_3$	-0.500839188	189.142413	3.07	4	cubic
67	$GUASnI_3$	-1.14038741	247.785908	1.7	5.9	cubic
68	$GUASnBr_3$	-0.88238027	209.851405	2.05	5	cubic
69	$GUASnCl_3$	-0.575766419	188.423884	2.7	4.1	cubic
70	$GUAGeI_3$	-1.227358376	239.675425	1.8	5.6	cubic
71	$GUAGeBr_3$	-0.931641337	204.834552	2.7	3.8	cubic
72	$GUAGeCl_3$	-0.750871863	181.380137	3.35	4.4	cubic
73	AZPbI3	-1.004791141	260.106806	1.33	7.21	cubic
74	$AZPbBr_3$	-0.930968134	222.864406	2.68	4.5	cubic
75	$AZPbCl_3$	-0.708045595	201.246225	3.16	3.94	cubic
76	$AZSnI_3$	-1.175418284	251.354111	1.35	5.9	cubic
77	$AZSnBr_3$	-1.003867267	221.544331	2.08	4.7	cubic
78	$AZSnCl_3$	-0.825861315	204.612222	2.7	3.9	cubic
79	$AZGeI_3$	-1.170090061	248.33598	1.85	5.4	cubic

80	AZGeBr3	-1.078569334	215.12522	2.56	4.43	cubic
81	AZGeCl3	-1.00483127	195.32461	3.24	3.7	cubic

S2. Clustering results of formation data from WEKA

See Run information in the file : Clusteringresultsofformationdata.text

S3. The classification results of the (trees.REPTree) classifier in Weka.

See Run information in the file : trees.REPTreeclassifierinWeka.txt

S4. Classification Learner App (Matlab)

The formation data splitting model

Open data from file

```
cd 'C:\Data'
```

TODO: Split the data into training and test sets

```
tbl = readtable('Formation energy_Band gap_Volume_constant dielectric.csv');
% Create the cvpartition variable (Reserve 20% of data for testing)
pt = cvpartition(tbl.structure,'HoldOut',0.2);
%Store training and test data with a new variable
idxTrain = training(pt);
idxTest = test(pt);
% Create the training and test tables
dataTrain = tbl(training(pt),:);
dataTest = tbl(test(pt),:);
% open The Classification Learner App
classificationLearner
%To make predictions on a new table, T:
yfit = trainedModel.predictFcn(dataTest);
 trainedModel.HowToPredict
%This script is to check the accuracy of the imported models
predictions = char(trainedModel.predictFcn(dataTest));
PredictedDAta = trainedModel.predictFcn(dataTest);
cat = categorical(tbl.structure);
%%accuracy
isocorrect = predictions == cell2mat(string((cat.structure)));
isocorrect=iscorrect(:,2);
accuracy = sum(iscorrect)*100/20;
```

S5. Table S2. Stability Data for 63 ABX_3 perovskites.

	Formula	*t (tolerance)*	*μ (octahedral)*
1	$MAPbI_3$	0.834352	0.540909091
2	$MAPbBr_3$	0.844047	0.607142857
3	$MAPbCl_3$	0.850893	0.657458564
4	$MASnI_3$	0.857107	0.5
5	$MASnBr_3$	0.868872	0.56122449
6	$MASnCl_3$	0.87721	0.607734807
7	$MAGeI_3$	0.965343	0.331818182
8	$MAGeBr_3$	0.988382	0.37244898
9	$MAGeCl_3$	1.004992	0.403314917
10	$DMAPbI_3$	1.026253	0.540909091
11	$DMAPbBr_3$	1.050569	0.607142857
12	$DMAPbCl_3$	1.067741	0.657458564
13	$DMASnI_3$	1.054242	0.5
14	$DMASnBr_3$	1.081468	0.56122449
15	$DMASnCl_3$	1.100764	0.607734807
16	$DMAGeI_3$	1.187372	0.331818182
17	$DMAGeBr_3$	1.23022	0.37244898
18	$DMAGeCl_3$	1.261112	0.403314917
19	$TMAPbI_3$	1.067971	0.540909091
20	$TMAPbBr_3$	1.095465	0.607142857
21	$TMAPbCl_3$	1.114882	0.657458564
22	$TMASnI_3$	1.097097	0.5
23	$TMASnBr_3$	1.127684	0.56122449
24	$TMASnCl_3$	1.149363	0.607734807
25	$TMAGeI_3$	1.235639	0.331818182
26	$TMAGeBr_3$	1.282793	0.37244898

27	$TMAGeCl_3$	1.31679	0.403314917
28	$EAPbI_3$	1.030425	0.540909091
29	$EAPbBr_3$	1.055058	0.607142857
30	$EAPbCl_3$	1.072456	0.657458564
31	$EASnI_3$	1.058528	0.5
32	$EASnBr_3$	1.086089	0.56122449
33	$EASnCl_3$	1.105624	0.607734807
34	$EAGeI_3$	1.192198	0.331818182
35	$EAGeBr_3$	1.235477	0.37244898
36	$EAGeCl_3$	1.26668	0.403314917
37	$FAPbI_3$	0.986622	0.540909091
38	$FAPbBr_3$	1.007917	0.607142857
39	$FAPbCl_3$	1.022958	0.657458564
40	$FASnI_3$	1.013529	0.5
41	$FASnBr_3$	1.037562	0.56122449
42	$FASnCl_3$	1.054595	0.607734807
43	$FAGeI_3$	1.141518	0.331818182
44	$FAGeBr_3$	1.180275	0.37244898
45	$FAGeCl_3$	1.208218	0.403314917
46	$GuaPbI_3$	1.038769	0.540909091
47	$GuaPbBr_3$	1.064038	0.607142857
48	$GuaPbCl_3$	1.081884	0.657458564
49	$GuaSnI_3$	1.067099	0.5
50	$GuaSnBr_3$	1.095333	0.56122449
51	$GuaSnCl_3$	1.115344	0.607734807
52	$GuaGeI_3$	1.201852	0.331818182
53	$GuaGeBr_3$	1.245992	0.37244898
54	$GuaGeCl_3$	1.277815	0.403314917
55	$AzPbI_3$	0.980364	0.540909091
56	$AzPbBr_3$	1.001183	0.607142857
57	$AzPbCl_3$	1.015886	0.657458564

58	$AzSnI_3$	1.007101	0.5
59	$AzSnBr_3$	1.03063	0.56122449
60	$AzSnCl_3$	1.047306	0.607734807
61	$AzGeI_3$	1.134278	0.331818182
62	$AzGeBr_3$	1.172389	0.37244898
63	$AzGeCl_3$	1.199866	0.403314917

S6. Cluster Data

Clustering results of stability data are in the file : Output_Clusteringresultsofstabilitydata.txt

S7. Table S3. The stability database with new attribute (cluster).

	Formula	t (tolerance)	μ (octahedral)	Cluster
1	$MAPbI_3$	0.834352	0.540909	cluster1
2	$MAPbBr_3$	0.844047	0.607143	cluster1
3	$MAPbCl_3$	0.850893	0.657459	cluster1
4	$MASnI_3$	0.857107	0.5	cluster1
5	$MASnBr_3$	0.868872	0.561224	cluster1
6	$MASnCl_3$	0.87721	0.607735	cluster1
7	$MAGeI_3$	0.965343	0.331818	cluster5
8	$MAGeBr_3$	0.988382	0.372449	cluster5
9	$MAGeCl_3$	1.004992	0.403315	cluster5
10	$DMAPbI_3$	1.026253	0.540909	cluster5
11	$DMAPbBr_3$	1.050569	0.607143	cluster4
12	$DMAPbCl_3$	1.067741	0.657459	cluster4
13	$DMASnI_3$	1.054242	0.5	cluster4
14	$DMASnBr_3$	1.081468	0.561224	cluster4
15	$DMASnCl_3$	1.100764	0.607735	cluster2
16	$DMAGeI_3$	1.187372	0.331818	cluster3

17	$DMAGeBr_3$	1.23022	0.372449	cluster3
18	$DMAGeCl_3$	1.261112	0.403315	cluster3
19	$TMAPbI_3$	1.067971	0.540909	cluster4
20	$TMAPbBr_3$	1.095465	0.607143	cluster4
21	$TMAPbCl_3$	1.114882	0.657459	cluster2
22	$TMASnI_3$	1.097097	0.5	cluster4
23	$TMASnBr_3$	1.127684	0.561224	cluster2
24	$TMASnCl_3$	1.149363	0.607735	cluster2
25	$TMAGeI_3$	1.235639	0.331818	cluster3
26	$TMAGeBr_3$	1.282793	0.372449	cluster3
27	$TMAGeCl_3$	1.31679	0.403315	cluster3
28	$EAPbI_3$	1.030425	0.540909	cluster5
29	$EAPbBr_3$	1.055058	0.607143	cluster4
30	$EAPbCl_3$	1.072456	0.657459	cluster4
31	$EASnI_3$	1.058528	0.5	cluster4
32	$EASnBr_3$	1.086089	0.561224	cluster4
33	$EASnCl_3$	1.105624	0.607735	cluster2
34	$EAGeI_3$	1.192198	0.331818	cluster3
35	$EAGeBr_3$	1.235477	0.372449	cluster3
36	$EAGeCl_3$	1.26668	0.403315	cluster3
37	$FAPbI_3$	0.986622	0.540909	cluster5
38	$FAPbBr_3$	1.007917	0.607143	cluster5
39	$FAPbCl_3$	1.022958	0.657459	cluster5
40	$FASnI_3$	1.013529	0.5	cluster5
41	$FASnBr_3$	1.037562	0.561224	cluster5
42	$FASnCl_3$	1.054595	0.607735	cluster4
43	$FAGeI_3$	1.141518	0.331818	cluster2
44	$FAGeBr_3$	1.180275	0.372449	cluster2
45	$FAGeCl_3$	1.208218	0.403315	cluster3
46	$GuaPbI_3$	1.038769	0.540909	cluster5
47	$GuaPbBr_3$	1.064038	0.607143	cluster4

48	$GuaPbCl_3$	1.081884	0.657459	cluster4
49	$GuaSnI_3$	1.067099	0.5	cluster4
50	$GuaSnBr_3$	1.095333	0.561224	cluster4
51	$GuaSnCl_3$	1.115344	0.607735	cluster2
52	$GuaGeI_3$	1.201852	0.331818	cluster3
53	$GuaGeBr_3$	1.245992	0.372449	cluster3
54	$GuaGeCl_3$	1.277815	0.403315	cluster3
55	$AzPbI_3$	0.980364	0.540909	cluster5
56	$AzPbBr_3$	1.001183	0.607143	cluster5
57	$AzPbCl_3$	1.015886	0.657459	cluster5
58	$AzSnI_3$	1.007101	0.5	cluster5
59	$AzSnBr_3$	1.03063	0.561224	cluster5
60	$AzSnCl_3$	1.047306	0.607735	cluster4
61	$AzGeI_3$	1.134278	0.331818	cluster2
62	$AzGeBr_3$	1.172389	0.372449	cluster2
63	$AzGeCl_3$	1.199866	0.403315	cluster3

S8. The trees.j48 classifier information

The run information is in the treesj48classifier information.txt

S9. Output of the functions.Logistic classifier information

The output is in the Outputofthefunctions.Logisticclassifierinformation.txt

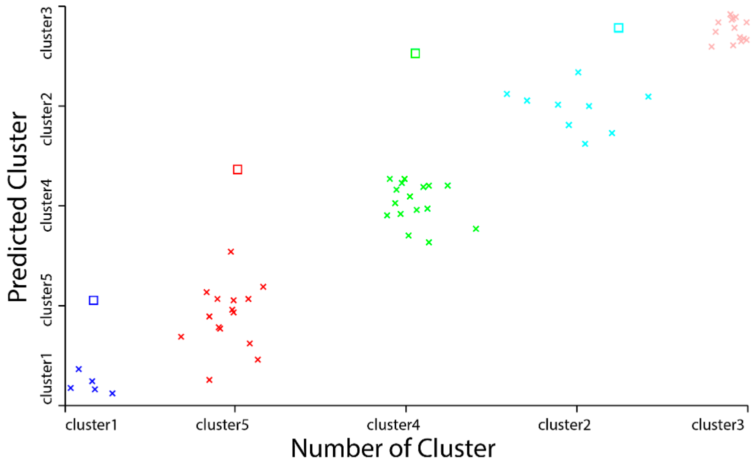

Figure S1. Visualize the trees.j48 classifier error, where the cubic points are the error classified cluster.

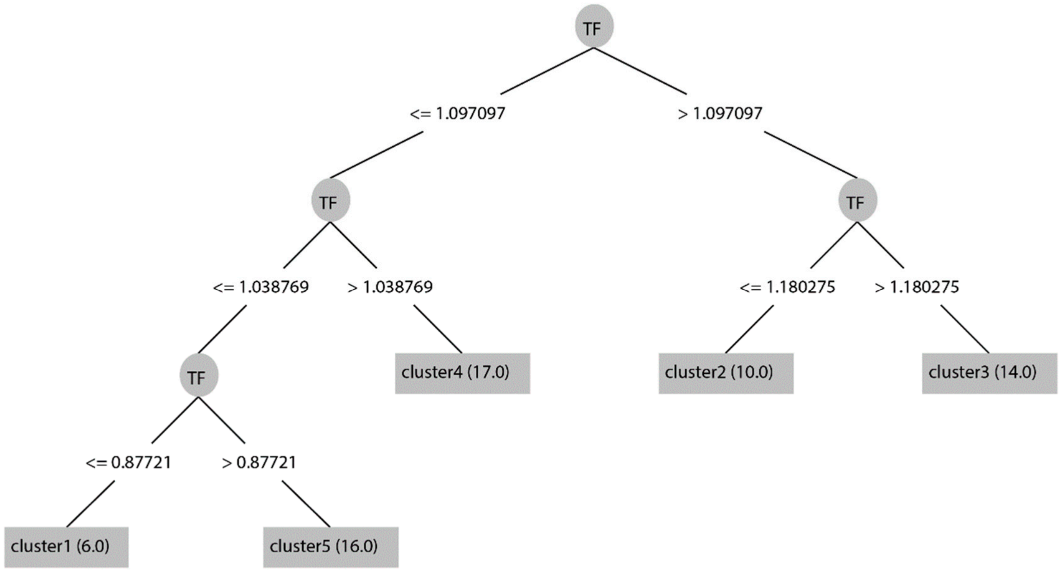

Figure S2. The tree for trees.j48 classifier.

S10. MATLAB code for using the conditions of the stability limits to identify the stable compounds

---

Open file

```
cd 'C:\Users\Stability Data'
StabilitydataCluster = readtable('Stability data with cluster attributes');
```

1. Change the name of attributes

```
StabilitydataCluster.Properties.VariableNames{2} = 'ToleranceFactor , t';
StabilitydataCluster.Properties.VariableNames{3} = 'OctahedralFactor , μ';
```

2. Adding a new variable that includes stability conditions

```
% if stabilityTF=0 or stabilityOcta=0 that mean it is in the unstable limits,
% If they =1  that mesn it is in the stable limit.
StabilityTF = StabilitydataCluster.("ToleranceFactor , t") > 0.81;
StabilityTF = StabilitydataCluster.("ToleranceFactor , t") < 1.11;
StabilityOcta = StabilitydataCluster.("OctahedralFactor , μ") > 0.89;
StabilityOcta = StabilitydataCluster.("OctahedralFactor , μ") > 0.44;

% If tolerance and octahedral factors in the stability limits, then stability=2
% If one of them in the stable but another one is in the instable, then stability=1
% If tolerance and octahedral factors in the instability limits, then stability=0

Stability= StabilityTF+StabilityOcta;

% converting from numric to nomenal
StabilityTF = nominal(StabilityTF);
StabilityOcta = nominal(StabilityOcta);
Stability = nominal(Stability);

% change name of value, If stablilityTF=true then it will be stableTF, if stablilityTF=false then 0
StabilityTF(StabilityTF=="true") = 't stable';
StabilityTF(StabilityTF=="false") = '0';

% change name of value, If stabilityTF=true then it will be stableTF, if stabilityTF=false then instableTF
StabilityOcta(StabilityOcta=="true") = 'μ stable';
StabilityOcta(StabilityOcta=="false") = '0';
```

```
nomStability = nominal(Stability);

Stability(Stability=="2") = 'stable';
Stability(Stability=="0") = 'non';
Stability(Stability=="1") = 'one Stable';

Stable = table(StabilityTF,StabilityOcta,Stability);

StabilityData = [StabilitydataCluster Stable];
```

3. Add stability compounds in new table

```
StabilityFormula = StabilityData.Formula(StabilityData.Stability=='stable');
TFCompound = StabilityData.("ToleranceFactor , t")(StabilityData.Stability=='stable');
OctaCompounds = StabilityData.("OctahedralFactor , μ")(StabilityData.Stability=='stable');
CCluster = StabilityData.('cluster')(StabilityData.Stability=='stable');

StabilityCompounds = table(StabilityFormula,TFCompound,OctaCompounds,CCluster);
```

4. Save final data as csv file

```
writetable(StabilityCompounds,'FinalStabilityValues.csv');
```

S11. Table S4. Final 38 compounds that achieved the stability conditions for the tolerance and octahedral factor ($0.81 < t < 1.11$, $0.44 < \mu < 0.89$) extracted from the main dataset.

	Formula	*t*	*μ*	*Cluster*
1	*'MAPbI₃'* →	0.834352	0.540909	'cluster1'
2	*'$MAPbBr_3$'*	0.844047	0.607143	'cluster1'
3	*'$MAPbCl_3$'*	0.850893	0.657459	'cluster1'
4	*'$MASnI_3$'*	0.857107	0.5	'cluster1'
5	*'$MASnBr_3$'*	0.868872	0.561224	'cluster1'
6	*'$MASnCl_3$'*	0.87721	0.607735	'cluster1'
7	*'$DMAPbI_3$'*	1.026253	0.540909	'cluster5'
8	*'$DMAPbBr_3$'*	1.050569	0.607143	'cluster4'

9	*'DMAPbCl$_3$'*	1.067741	0.657459	'cluster4'
10	*'DMASnI3'*	1.054242	0.5	'cluster4'
11	*'DMASnBr$_3$'*	1.081468	0.561224	'cluster4'
12	*'DMASnCl$_3$'*	1.100764	0.607735	'cluster2'
13	*'TMAPbI$_3$'*	1.067971	0.540909	'cluster4'
14	*'TMAPbBr$_3$'*	1.095465	0.607143	'cluster4'
15	*'TMASnI$_3$'*	1.097097	0.5	'cluster4'
16	*'EAPbI$_3$'*	1.030425	0.540909	'cluster5'
17	*'EAPbBr$_3$'*	1.055058	0.607143	'cluster4'
18	*'EAPbCl$_3$'*	1.072456	0.657459	'cluster4'
19	*'EASnI$_3$'*	1.058528	0.5	'cluster4'
20	*'EASnBr$_3$'*	1.086089	0.561224	'cluster4'
21	*'EASnCl$_3$'*	1.105624	0.607735	'cluster2'
22	*'FAPbI$_3$'*	0.986622	0.540909	'cluster5'
23	*'FAPbBr$_3$'*	1.007917	0.607143	'cluster5'
24	*'FAPbCl$_3$'*	1.022958	0.657459	'cluster5'
25	*'FASnI$_3$'*	1.013529	0.5	'cluster5'
26	*'FASnBr$_3$'*	1.037562	0.561224	'cluster5'
27	*'FASnCl$_3$'*	1.054595	0.607735	'cluster4'
28	*'GuaPbI$_3$'*	1.038769	0.540909	'cluster5'
29	*'GuaPbBr$_3$'*	1.064038	0.607143	'cluster4'
30	*'GuaPbCl$_3$'*	1.081884	0.657459	'cluster4'
31	*'GuaSnI$_3$'*	1.067099	0.5	'cluster4'
32	*'GuaSnBr$_3$'*	1.095333	0.561224	'cluster4'
33	*'AzPbI$_3$'*	0.980364	0.540909	'cluster5'
34	*'AzPbBr$_3$'*	1.001183	0.607143	'cluster5'
35	*'AzPbCl$_3$'*	1.015886	0.657459	'cluster5'
36	*'AzSnI$_3$'*	1.007101	0.5	'cluster5'
37	*'AzSnBr$_3$'*	1.03063	0.561224	'cluster5'
38	*'AzSnCl$_3$'*	1.047306	0.607735	'cluster4'

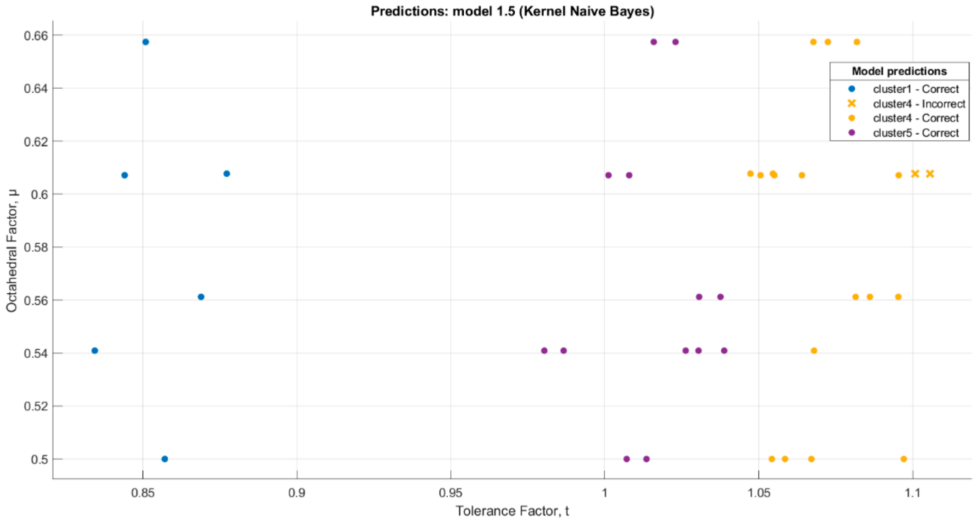

Figure S3. The Kernel Naive Bayes classifier prediction model for the 38 stable datasets.

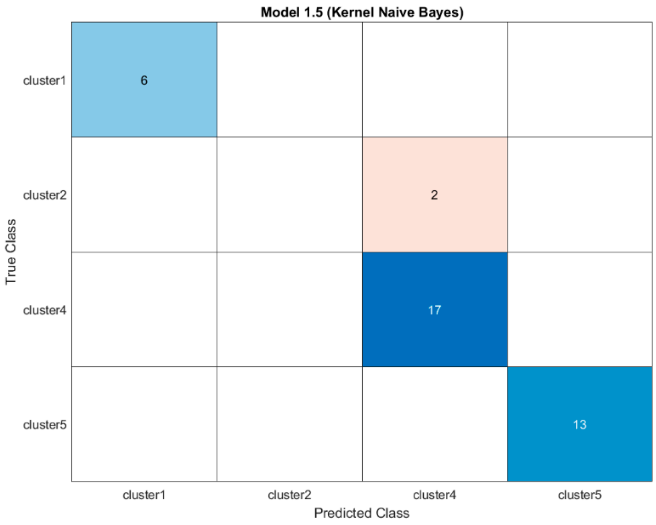

Figure S4. The confusion matrix of the Kernel Naive Bayes classifier for the 38 stable datasets.
